# Editorial: The Chemistry of Biofilms and Their Inhibitors

**DOI:** 10.3389/fchem.2020.00746

**Published:** 2020-08-20

**Authors:** Sérgio F. Sousa, Manuel Simões, Nenad Filipović

**Affiliations:** ^1^UCIBIO@REQUIMTE, BioSIM, Departamento de Biomedicina, Faculdade de Medicina, Universidade do Porto, Porto, Portugal; ^2^LEPABE, Department of Chemical Engineering, Faculty of Engineering, University of Porto, Porto, Portugal; ^3^Department of Chemistry and Biochemistry, Faculty of Agriculture, University of Belgrade, Belgrade, Serbia

**Keywords:** biofilms, virtual screening, antibiotics, antimicrobials, bacterial infections

Over the past few years, biofilm research has moved to the spotlight, resulting in a strong increase in the researchers studying such highly structured microbial community and in the number of articles published per year. The large body of work made available enabled the broadening of research in this field to new disciplines and methods, moving from the cellular level to new complementary perspectives that tackle the problem from a molecular and atomic level—the chemical perspective—focusing on how the molecules involved interact, the bonds formed and their stability, the specific conformations adopted, etc. At the moment, a number of molecular targets to counteract biofilm formation and development as well as synthetic and natural inhibitors are known. For some known antibiofilm agents, the mechanisms of action are still unknown. The future development in the field of antibiofilm agents needs to be shifted to computational design and synthesis of novel multi-targeting compounds as a more effective strategy for the treatment of multi-factorial diseases.

This special issue “The Chemistry of Biofilms and Their Inhibitors” compiles four original articles and five reviews outlining the most innovative researches regarding the chemistry of biofilms and chemical innovations to understand biofilm formation and promote its inhibition.

Soukarieh et al. reported the synthesis and biological evaluation of a new series of potent Pseudomonas quinolone signal regulator (PqsR) antagonists able to inhibit planktonic and biofilm grown of different strains of *Pseudomonas aeruginosa*. The work involved the hit-to-lead optimization, following an initial virtual screening of the University of Nottingham Managed Chemical Compound Collection (85,061 compounds), using OpenEye docking and *in vitro* screening of the top 500 results. The optimized compound showed very high inhibition activity against *P. aeruginosa* pyocyanin production and Pseudomonas quinolone signal (Pqs) system signaling, in both planktonic cultures and biofilms.

The contribution by Vogel et al. shows that the immobilization of acyclase PvdQ (an N-terminal nucleophile hydrolase that is a part of the pyoverdine gene cluster, pvd) on polydimethylsiloxane silicone (PDMS) creates a surface with quorum quenching properties that significantly reduces biofilm formation by *P. aeruginosa*. These results suggest this as promising strategy to control infections by minimizing the colonization of indwelling medical devices such as urinary or intravascular catheters.

The contribution by Walsh et al. reports the antimicrobial activity of a variety of naturally occurring phenols and their derivatives, against *Staphylococcus epidermidis* and *P. aeruginosa* present in biofilm and in the planktonic state. In particular, the authors have evaluated thymol, carvacrol and eugenol, and their allyl, 2-methallyl and propyl derivatives. The results demonstrated that for the bacteria in the planktonic state, the presence of an allyl group leads to an increase in potency for both thymol and carvacrol. However, the parent compounds exhibited higher activity than their derivatives against bacteria in biofilms. A similar effect was observed for guaiacol/eugenol molecules, with the larger molecule (eugenol) exhibiting higher activity toward the bacteria in the planktonic state while the smaller one (guaiacol), displays higher activity in biofilms. The results stress the importance of performing biofilm assays to develop structure-activity relationships when the objective is to target biofilms.

The contribution of Mahmoud et al. reports the development and characterization of a rapid-release platform, composed of polymeric electrospun fibers (EF) that encapsulate a peptide (BAR, SspB Adherence Region), previously developed by the authors and with confirmed antibiofilm activity *in vivo* and *in vitro*. The study reports also the evaluation of fiber safety and functionality against *Porphyromonas gingivalis*/*Streptococcus gordonii* biofilms *in vitro*, suggesting that BAR-incorporated EFs may provide a safe and specifically-targeted rapid-release platform to inhibit and disrupt dual-species biofilms in the oral cavity.

Li et al. presented a contribution on the application of cascade reactions involving reactive oxygen species in the control of bacterial infections. In this perspective, the authors reported the potential usefulness of cascade reactions as a new infection control strategy, while stressing the vast amount of work that still remains to be done in this field. In particular, the authors highlighted the need to increase the bacterial killing efficacy to clinically effective levels, preferentially by using endogenously available substrates.

Verderosa et al. contribution reviews the current status of bacterial biofilm eradication agents. In particular, the authors focus on the current understanding of biofilm antibiotic tolerance mechanisms, providing also an overview of biofilm remediation strategies and concentrating on the most promising biofilm eradication agents and approaches.

Wang et al. contribution focus on the current status on lipid-based antimicrobial delivery-systems for the treatment of bacterial infections. In this review, the authors focus on antimicrobial nanocarriers, including micelles and liposomes, at different levels of complexity and sophistication, describing the different types, preparation strategies, and their application in the treatment of infectious biofilms. Special emphasis is dedicated to the traditional problems faced in the antimicrobial treatment of infectious biofilms and the advantages offered by liposomal antimicrobial nanocarriers.

The contribution by Cho et al. reviews the currently identified inhibitors of diguanylate cyclase that interfere with bacterial biofilm inhibition, including natural molecules, c-di-GMP analogs, GTP analogs and small synthetic molecules. Particular attention is dedicated to their mode of action, the methods of high-throughput screening and assay involved in their discovery and the chemical libraries used in screening.

Qvortrup et al. contributed with a review dedicated to small molecules with anti-biofilm activity developed on the basis of a molecular understanding of the mechanisms involved in the formation and dispersion of biofilms. Special emphasis is given to pilicides and curlicides, inhibiting the initial steps of biofilm formation by *Escherichia coli*; compounds interfering with c-di-GMP signaling in *P. aeruginosa* and *E. coli*; as well as compounds that inhibit quorum-sensing in *P. aeruginosa* and *Acinetobacter baumannii*.

It has been a pleasure to participate in the edition of this exciting topic of Frontiers in Chemistry. The issue brings together a wide variety of articles and reviews focusing on biofilms, from a chemical perspective. The editors hope that the articles will be of interest to researchers in the field of medicinal and pharmaceutical chemistry, particularly those working with biofilms.

## Author Contributions

All authors listed have made a substantial, direct and intellectual contribution to the work, and approved it for publication.

## Conflict of Interest

The authors declare that the research was conducted in the absence of any commercial or financial relationships that could be construed as a potential conflict of interest.

